# Resveratrol from Dietary Supplement to a Drug Candidate: An Assessment of Potential

**DOI:** 10.3390/ph15080957

**Published:** 2022-08-01

**Authors:** Shivani Khattar, Sauban Ahmed Khan, Syed Amir Azam Zaidi, Mahdi Darvishikolour, Uzma Farooq, Punnoth Poonkuzhi Naseef, Mohamed Saheer Kurunian, Mohammed Zaafar Khan, Athar Shamim, Mohd Masih Uzzaman Khan, Zeenat Iqbal, Mohd. Aamir Mirza

**Affiliations:** 1 Department of Pharmaceutics, School of Pharmaceutical Education and Research, Jamia Hamdard, New Delhi 110062, India; sk06011998@gmail.com (S.K.); uzma411@gmail.com (U.F.); atharshamim@gmail.com (A.S.); 2 School of Pharmaceutical Education and Research, Jamia Hamdard, New Delhi 110062, India; saubankhan99@gmail.com (S.A.K.); amirazamzaidi1@gmail.com (S.A.A.Z.); mahdidarvishi19@gmail.com (M.D.); zaafarkhan14@gmail.com (M.Z.K.); 3 Department of Pharmaceutics, Moulana College of Pharmacy, Perinthalmanna, Kerala 679321, India; drnaseefpp@gmail.com; 4 Department of Dental Technology, College of Applied Medical Sciences, King Khalid University, Abha 61421, Saudi Arabia; mkurunian@kku.edu.sa; 5 Department of Pharmaceutical Chemistry and Pharmacognosy, Unaizah College of Pharmacy, Qassim University, Unaizah 51911, Saudi Arabia; mo.khan@qu.edu.sa

**Keywords:** neuroprotective, anti-thrombogenic, antioxidants, cardioprotective, carcinogenic and anti-aging

## Abstract

Resveratrol (RVT) is a well known phyto-chemical and is widely used in dietary supplements and botanical products. It shows a wide range of pharmacological/beneficial effects. Therefore, it can be a potential candidate to be developed as phyto-pharmaceutical. Multiple diseases are reported to be treated by the therapeutic effect of RVT since it has antioxidant, anti-cancer activity and anti-inflammatory activities. It also has a major role in diabetes, arthritis, cardiac disorder and platelet aggregation etc. The major requirements are establishments regarding safety, efficacy profile and physicochemical characterization. As it is already being consumed in variable maximum daily dose, there should not be a major safety concern but the dose needs to be established for different indications. Clinical trials are also being reported in different parts of the world. Physicochemical properties of the moiety are also well reported. Moreover, due to its beneficial effect on health it leads to the development of some intellectual property in the form of patents.

## 1. Introduction

Resveratrol (3, 4′, 5-trihydroxystilbene) is a stilbenoid class of compound and works as phytoalexin (i.e., a substance that is produced by plant tissues in response to contact with a parasite and specifically inhibits the growth of that parasite) [1]. Because of its intriguing pharmacological potential, it has recently gained a lot of study attention. Early studies have illustrated the presence of substantial amounts of Resveratrol (RVT) in wounded, infected, and UV-treated leaves [2]. It is primarily found in grapes, peanuts, and berries. In the 1940s, RVT was discovered in the white hellebore plant. It is also found in processed plant products; its presence in red wine (concentrations of 0.1–14.3 mg/L) has been proposed as a possible explanation for the “French paradox,” the observation of an unusually low rate of heart disease among Southern French people who drink a lot of red wine, despite a high saturated fat diet [3,4]. SIRT1, one of the mammalian versions of the sirtuin family of proteins, is activated by RVT [5], deacetylates histones and non-histone proteins, such as transcription factors [6]. Metabolism, stress resistance, cell survival, cellular senescence, inflammation-immune function, endothelial functions, and circadian rhythms are all affected by the SIRT1-regulated pathway [5]. Since RVT has been demonstrated to activate SIRT1, it is expected to help people with disorders such as improper metabolic regulation, inflammation, and cell cycle abnormalities. RVT’s usage as a nutraceutical and a therapeutic agent for a variety of disorders has been extensively investigated in preclinical trials as a natural molecule. Moreover, clinical trials are been conducted globally so as to establish its therapeutic efficacy for treatment of different diseases. A detailed description of it is mentioned in subsequent subtopics.

Because of the substantial dangers associated with standard cancer therapies, such as surgery and chemotherapy, its usage is of particular interest to cancer patients [7]. The intricacies of cancer cell signalling networks make it difficult for targeted inhibitors that target only one network to be effective. RVT, on the other hand, has been found to have chemo-preventive and chemotherapeutic effects on tumours in vitro and in vivo by targeting various pathways, making it a promising anticancer drug [8]. RVT has an effect on carcinogenesis at all three stages: initiation, promotion, and progression. Additionally, RVT has been demonstrated to directly trigger the apoptotic pathway via a variety of methods [9,10]. For example it has an effect on the nuclear factor κB (NF-B) signalling system, which governs inflammation, immunological response to infection, and cellular response to stimuli [11]. Furthermore, it has also been demonstrated that it blocks the IGF-1R/Akt/Wnt pathways and activates p53, influencing cell development and carcinogenesis [12]. Moreover, it can also block the PI3K/Akt pathway, which regulates cell differentiation, development, and proliferation, and other factors [13]. Several studies have been conducted to investigate the various methods by which RVT operates the PI3K/Akt pathway.

Considering its diverse potentials, an attempt has been made to advocate the development of RVT as a drug molecule. Although there are several dietary supplement products available in the global market, a thorough investigation in terms of clinical safety and efficacy may pave the way for a new therapeutic molecule.

Since resveratrol is a well-known antioxidant and has been utilised as a nutraceutical for many years, it possesses a range of therapeutic properties. As some of its clinical trials are completed, it could therefore be regarded as a promising drug candidate to be used in the treatment of certain diseases. 

## 2. Occurrence/Sources

**Figure 1:**
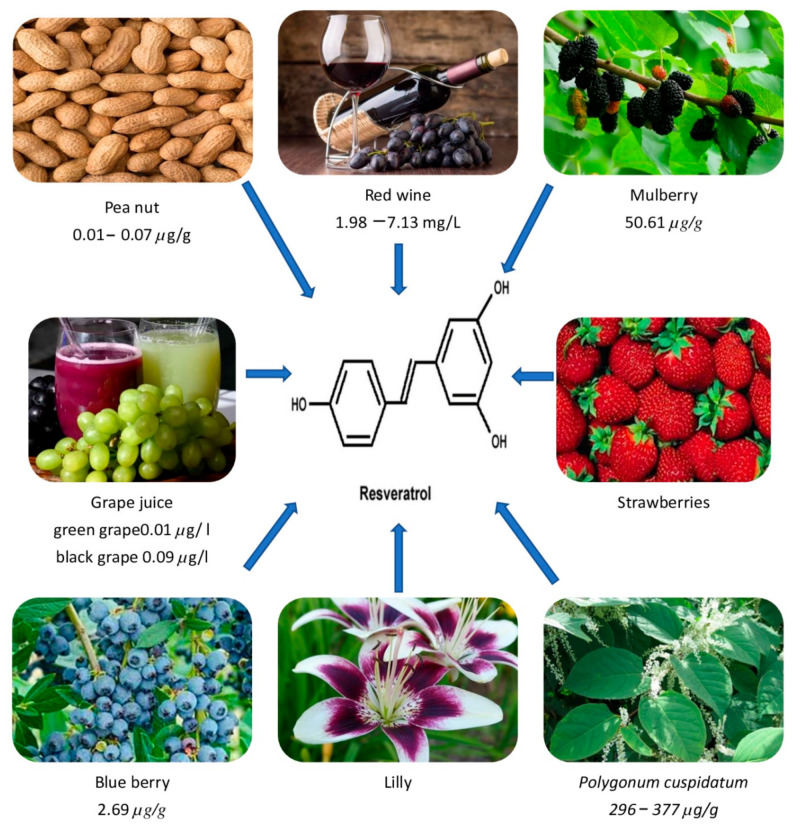
shows the occurrence of RVT in different sources.

Dark grape extracts (*Vitis vinifera*) and giant knot weed (*Polygonnum cuspidatum*, a perennial shrub) are the richest natural sources of RVT (Table 1). It is also abundant in labrusca and muscadine grapes. Additionally, it is also found in plants such as eucalyptus, spruce, and lily, as well as in foods such as mulberries, peanuts, blueberries, strawberries, hops, and their derivatives [14,15]. It may also be found in the vines, roots, seeds, and stalks, but the skin has the highest concentration, with 50–100 g per gm [16]. RVT is a phytoalexin, which is a kind of antibiotic molecule generated by plants as part of their defense against diseases. For example, in reaction to an invading fungus, RVT is produced from p-coumaroyl CoA and malonyl CoA [1,17]. As fungal infections are more prevalent in cooler areas, grapes produced in cooler climates have a higher quantity of RVT [18]. Table 2 shows the total RVT concentration of several wines and foods. Similarly, Figure 1 shows the occurrence of RVT in different sources.

## 3. Physiological Effects of RVT

**Figure 2:**
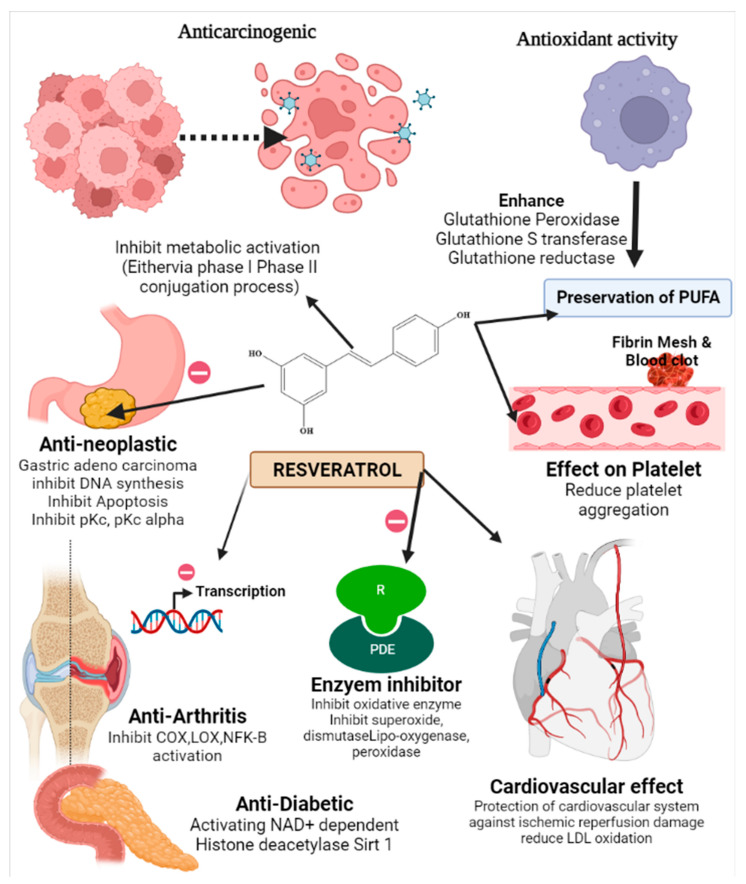
shows the mechanism of action of resveratrol.

RVT has different physiological effects. It has been demonstrated to have neuroprotective benefits at low doses which is usually present in food [36] as well as positive effects on the cardiovascular system [37]. These benefits are mostly due to its anti-oxidant effect. Recently, it has been studied for its potential health advantages in various medical fields, such as anticancer activities when provided at larger, non-physiological concentrations. In the previous research, 500 mg of RVT per day is the bare minimum quantity required to protect against cancer [38]. Red wine is a rich source of RVT having approximately 1.98–7.13 mg/L of RVT. In red wine grapes, the terminal enzyme that is involved in biosynthesis of resveratrol is the stilbene synthase, which is activated by exogenous stress factors, UV light, and defined chemical signals from pathogenic fungi [39]. Therefore, the content of resveratrol and its isomers in the final wine products may significantly differ between countries and cultivation areas. Resveratrol can modulate the activity of SIRT1, a critical deacetylase that impacts the acetylation status of p53, forkhead proteins, and DNA repair enzymes [40]. On the other hand, activation of SIRT1 by resveratrol treatment reduced tumorigenesis in a mouse model. As a consequence, the binding of resveratrol to SIRT1 is associated with a signal that mimics calorie restriction and increases DNA stability [41]. It is reported to inhibit proliferation and induces apoptotic cell death in multiple cancer cell types in vitro [42]. Additionally, it has been shown to inhibit angiogenesis and delay tumour growth, impede carcinogenesis and reduce experimental carcinogenesis [43] in animal models of cancer. It affects the carcinogenesis process by influencing the three phases: tumor initiation, promotion, and progression, as well as suppressing the decisive stages of carcinogenesis, namely angiogenesis and metastasis [9]. It can also induce apoptosis, stop the cell cycle, and inhibit kinase pathways (Figure 2). Other remarkable activities are anti-thrombogenic, anti-inflammatory, cardioprotective, neuroprotective and anti-aging. Role of RVT in the human body is given below and its detailed mechanism of action for different condition is mentioned in Table 3.

### 3.1. Antioxidants

One of the most useful findings has been the antioxidant action of RVT. Because of its capacity to stimulate the activity of a range of antioxidant enzymes, RVT is both a free radical scavenger and a powerful antioxidant. The capacity of polyphenolic compounds to behave as antioxidants is dependent on the redox characteristics of their phenolic hydroxy groups and the possibility for electron delocalization across the chemical structure (Alarco et al., 2006), highlighting the significance of RVT as a natural antioxidant, proposing three possible antioxidant mechanisms: (i) competing with coenzyme Q and decreasing the oxidative chain complex, which is the location of ROS formation, (ii) scavenging O_2_ free radicals generated in the mitochondria, and (iii) suppression of LP (lipid peroxidation) induced by Fenton reaction products. Several investigations have showed that RVT may scavenge both O_2_ and OH free radicals [53,55]. In contrast, [56] found that RVT had no effect on XO (Xanthine Oxidase) activity or scavenged O_2_ free radicals in rat macrophage extracts utilising the enzymatic hypoxanthine oxidase–XO (xanthine oxidase) system. RVT can keep the concentration of intracellular antioxidants in biological systems stable. Stilbene, for example, protects the glutathione concentration in peripheral blood mononuclear cells against oxidative damage produced by 2-deoxy-D-ribose [57]. RVT significantly reduced the oxidation of protein thiol groups in human blood platelets [58]. Likewise, RVT increased glutathione levels in human lymphocytes stimulated with H_2_O_2_ in a concentration-dependent way. In another research it was found that RVT raised the levels of many antioxidant enzymes, including glutathione peroxidase, glutathione S-transferase, and glutathione reductase [53]. RVT’s antioxidant capability for the protection of polyunsaturated fatty acids (PUFA) has been described by [54].

### 3.2. Platelet Aggregation

RVT has been shown to have anti-platelet action [59]; however, the exact mechanisms are yet unknown. In a recent study, protein kinase C inhibitor (PKCI) and RVT (RSVL) exhibited an additive impact in decreasing platelet aggregation content, as did platelet membrane bound fibrinogen (PFig) [60,61]. Moreover, RSVL (at a concentration around 50 M) significantly reduced PKC activity in platelet membranes and the proportion of membrane PKC activity in total PKC activity [52]. In another investigation, RVT (0.05–0.25 μmol/L) inhibited platelet aggregation triggered by collagen (1 microg/mL) more effectively than other agonists [62]. In another study, RVT, at 10–1000 μmol/L concentrations, was found to be effective in preventing platelet aggregation in vitro produced by collagen, thrombin, and ADP in healthy people [63].

### 3.3. Enzyme Inhibitors

Trans-RVT inhibits oxidative enzymes in an animal cell system [64]. It inhibits superoxide dismutase, lipoxygenase, catalase, peroxidase, polyphenol oxidase, and 1-aminocyclopropane-1- carboxylic acid oxidase. Trans-RVT also inhibits lipoxygenase activity more efficiently than other lipoxygenase inhibitors such as propyl gallate, ibuprofen, ursolic acid, acetylsalicylic acid, and salicyl-hydroxamic acid [48]. The rate of inhibition rises as trans-RVT concentration increases. RVT, which has antioxidant action, suppresses matrix metalloproteinase via SIRT1 regulation in human fibrosarcoma cells, indicating that RVT might be a possible option for cancer chemoprevention [65]. According to a recent study, RVT (RSV) particularly inhibits inducible nitric oxide formation (iNOS) formation in muscle via a mechanism involving AMP-activated protein kinase (AMPK) but not deacetylase enzyme (SIRT1) activation [66]. RSV’s anti-inflammatory activity most likely adds to the plant polyphenol’s medicinal impact [67]. In another study, RVT was shown to suppress neuronal apoptosis and increase Ca2+/calmodulin-dependent protein kinase II activity in the diabetic mouse retina [68]. It was inferred that RVT obstructs diabetes-induced RGC mortality through down regulating calmodulin-dependent protein kinase II (CaMKII), suggesting that RVT may have potential therapeutic implications for the prevention of diabetes-induced visual impairment (Kim et al., 2010). RVT, a polyphenol found in red wine, prevents pancreatic cancer by blocking leukotriene A4 hydrolase [69]. It has a greater inhibitory impact than bestatin, a well-known inhibitor of LTA(4).

### 3.4. Anti-Carcinogenic Agents

RVT has been found to suppress carcinogenesis by influencing several molecular processes during the initiation, promotion, and progression phases [70]. RVT’s anti-initiation effect has been connected to the inhibition of metabolic activation and/or promotion of carcinogen detoxification via modulation of enzymes implicated in either phase I (i.e., cytochrome P450 enzymes (CYP)) or phase II conjugation processes. Several in vitro investigations have revealed that RVT suppresses the activity of the CYP1A1 and CYP1A2 enzymes [49]. The molecular processes responsible for RVT’s cancer-preventive impact might be the modulation of enzyme systems involved in carcinogen activation and detoxification. As these enzymes are also involved in drug metabolism, they may have an impact on therapeutic effectiveness and toxicity. A large number of in vitro investigations demonstrated that RVT affects cell proliferation, inflammation, apoptosis, angiogenesis, invasion, and metastasis through various intracellular targets. These include tumour suppressors p53 and Rb; cell cycle regulators cyclins [71], CDKs, p21WAF1, p27KIP, and INK, as well as the checkpoint kinases ATM/ATR; transcription factors NF-kappa B, AP-1, c-Jun, and c-Fos; angiogenic and metastatic factors VEGF and matrix metalloprotease 2/9; cyclooxygenases (L). In addition to its efficient antioxidant effects, there is mounting evidence that RVT has pro-oxidant activity under specific experimental settings, inducing oxidative DNA damage that may result in cell cycle arrest or apoptosis [72]. A recent study demonstrated for the first time that RVT has anti-proliferative, DNA damaging, and apoptotic effects in HNSCC cells independent of Smad4 status, both in vitro and in vivo, implying that more research is needed to establish its potential utility against head and neck squamous cell carcinoma (HNSCC) [73].

### 3.5. Anti-Neoplastic and Phytogenic Agent

RVT possess anti-cancer properties in tests that mimicked the three important phases of carcinogenesis. It has manifested to suppress cancer development and progression [74]. It functions as a selective oestrogen receptor modulator (SERM) and controls proteins involved in DNA synthesis and cell cycle regulation. RVT also has an effect on the activity of transcription factors involved in proliferation and stress responses, including NF-kB, AP1, and Egr1 [75].

### 3.6. Anti-Arthritic Agent

Arthritis is a chronic disease caused by the dysregulation of pro-inflammatory cytokines (e.g., tumour necrosis factor and interleukin-1beta) and proinflammatory enzymes that mediate the production of prostaglandins (e.g., cyclooxygenase-2) and leukotrienes (e.g., lipoxygenase), along with the expression of adhesion molecules, matrix metalloprotein and hyper-proliferation of synovial fibroblast [76]. The activation of the transcription factor nuclear factor-kappa B regulates all of these factors. Thus, any drug that may inhibit the production of tumour necrosis factor-alpha, interleukin-1beta, cycloxygenase-2, lipoxygenase, matrix metalloproteinases, or adhesion molecules, or inhibit the activation of NF-kappa B, has the potential to treat arthritis [50]. It was found that RVT acts as an inhibitor or mediator for several of these substances in our bodies.

### 3.7. Cardiotonic

RVT is a phytoestrogen, a powerful antioxidant, a scavenger of reactive oxygen species, and a metal chelator [77]. Thus, RVT may benefit the cardiovascular system by protecting it from ischemic-reperfusion injury [51]; it may also preserve and maintain the intact endothelium, it has anti-atherosclerotic effects, reduces LDL oxidation, suppresses platelet aggregation, and has estrogen-like activity [78]. As a result, RVT is potentially effective for cardiovascular disease.

### 3.8. Anti-Diabetic Potential

It has been established that RVT prolongs the life span of lower creatures by activating the NAD (+)-dependent histone deacetylase Sirt1 [79]. It was also discovered that RVT also boost lifespan and glucose homeostasis in mice via activating Sirt1-mediated deacetylation of the transcriptional coactivator PGC-1alpha [54]. In 2001, RVT (5–35 micromole/L) has been shown to elicit concentration-dependent relaxation of mesenteric arteries precontracted with nor-adrenaline (8 micromole/L) or KCl (125 mmol/L) in both lean and dietary obese rats [80]. Hyperglycemia, a characteristic of diabetes mellitus, causes hyper-osmotic response in vascular endothelial cells and leukocytes. Apoptotic cell death is frequently caused by hyper-osmotic shock. Because of its antioxidant properties, RVT has been shown to reduce high glucose-induced apoptotic alterations. Diabetes-related nephropathy is a severe vascular consequence and one of the leading causes of end-stage renal failure. Increased oxidative stress is a foremost contributor to the pathogenesis of diabetic nephropathy. In diabetic mice, RVT treatment dramatically reduced renal impairment and oxidative stress [81]. When insulin production by the islets of Langerhans is depleted, the majority of type 2 diabetes mellitus patients become insulin-dependent. In streptozotocin-induced diabetic rats, RVT was discovered to have hypoglycemic and hypolipidemic effects. When RVT was administered to diabetic rats on day 14 it was observed that the plasma glucose concentration was lowered by 25.3 percent, and the triglyceride concentration was reduced by 50.2 percent when compared to the placebo-treated rats, whereas in nicotinamide-treated diabetic rats on day 14, the plasma glucose concentration was lowered by only 20.3 percent, but the triglyceride concentration was reduced by 33 percent. RVT treatment significantly reduced insulin secretion and delayed the emergence of insulin resistance in STZ-nicotinamide DM rats [82].

### 3.9. Dermal Health

When administered topically, RVT cream decreased the production of HSV-1 lesions in the skin of mice. In addition to it, RVT cream also reduced herpes simplex virus (HSV) replication in the vagina of mice and restricts extravaginal diseases. As a result, RVT cream may possess some potential advantages for skin health, but additional research is needed to back up this health benefit claim.

### 3.10. Weight Management

RVT increases metabolism, allowing users to burn more calories throughout the day. As a result, RVT has weight loss advantages. When RVT was combined with genistein and quercetin adipogenesis was reduced in mouse and human adipocytes [42]. In contrast, one in vivo study illustrated that phytochemicals such as RVT, when combined with vitamin D, reduced weight gain and bone loss in a postmenopausal rat model [71]. A high-dose therapy (dosage: vitamin D + 400 mg/kg RVT + 2000 mg/kg quercetin + 1040 mg/kg genistein) decreased the body weight and fat pad weights in another investigation performed on elderly ovariectomized female rats. This medication significantly enhanced the serum levels of insulin-like growth factor-1 as well as of femoral bone mineral content. As a result, the synergistic effects of RVT and vitamin D may be useful in preventing bone loss and weight gain after menopause [71]. According to some studies, RVT may help prevent “weight gain” under specific situations. However, there is no conclusive proof that RVT aids in weight loss.

### 3.11. Anti-Inflammatory Activity

RVT manifests anti-inflammatory effects by modulating enzymes and pathways that produce inflammatory mediators, as well as inducing programmed cell death in activated immune cells. Even at high dosages, RVT has been demonstrated to have no negative side effects. As a result, RVT could be used either as complementary to or an alternative treatment for cancer and inflammatory illnesses [83].

### 3.12. Other Pathological/Physiological Conditions

Since microcirculation blockage and cytokine overproduction are implicated in many disorders, including acute pancreatitis, RVT as a platelet and cytokine inhibitor may aid acute pancreatitis patients [59]. In a Wistar rat investigation, RVT exhibits immunosuppressive properties as well as a protective impact on hepatocytes during allograft rejection [59]. RVT has been found to prevent ischemia perfusion (I/R) damage in the rat kidney through both antioxidant and anti-inflammatory pathways [84]. In one trial, researchers gave trans-RVT at a dose of 20 mg/kg/day for 90 days. Compared with a control group, the diameter of the seminiferous tubules was dramatically reduced from 437.5 ± 0.1 mum to 310.9 ± 0.1 mum. A considerable rise in tubular density accompanied this drop. The RVT-treated rats had considerably higher sperm counts than the control group, but sperm quality did not vary [85].

## 4. Pharmacokinetic Properties of RVT

### 4.1. Absorption and Bioavailability

RVT bioavailability in both rodents and humans reveals that this polyphenol possesses high oral absorption but rapid and widespread metabolism with no deleterious effects, resulting in only trace levels of unaltered RVT in the systemic circulation [86,87]. About 70% of orally administered RVT (25 mg) is rapidly (<30 min) absorbed and metabolized in humans, with a peak plasma level of 2 M of RVT metabolites and a half-life of 9–10 h [88]. Furthermore, drug absorption and metabolism processes differ significantly from person to person. The ability of the human colon to absorb and metabolize RVT is determined by hepatic function and local intestinal microflora metabolic activity [89].

### 4.2. Distribution

#### 4.2.1. Blood Transport

The affinity of a therapeutic agent to bind to protein transporters is typically linked to its efficacy [64]. Because RVT is poorly soluble in water, it must be linked to plasma proteins in order to be distributed throughout the body and bioavailable [90]. Indeed, RVT can attach to serum proteins such as lipoproteins, hemoglobin, and albumin during its transport, facilitating carrier-mediated cellular absorption and then passively diffusing through the plasma membrane [91]. Researchers examined RVT’s binding capabilities to plasma proteins such as human serum albumin (HSA) and hemoglobin (Hb) and found that both complexes formed are spontaneous and exothermic. The RVT–HSA complex has a greater binding constant than RVT–Hb, indicating that HSA has a stronger affinity for RVT [92]. Hydrophobic interactions appear to be important in RVT’s binding to the hydrophobic cavity of HSA, whereas hydrogen bonding is the main force that works in RVT’s binding to the core cavity of Hb where certain residues engage directly with the compound’s hydroxyl groups. Electrostatic interactions can also be involved in the formation of both complexes since residues with positive charge are in the proximity of the binding compound.

#### 4.2.2. Liver Uptake

The liver is known to play an important role in RVT bioavailability. After oral dosing, rats and mice accumulate the most RVT in their livers [93,94]. Despite this, no toxicity or hepatocyte lyses were found after high-dose RVT administration, which is significant because certain anti-neoplastic drugs produce hepatotoxicity, which limits their efficacy in anticancer therapy [95]. Furthermore, RVT’s high absorption by liver cells, combined with its low toxicity, shows that it plays a significant role in the prevention of liver disorders. In addition to passive diffusion, it was discovered that RVT reaches the liver cells through an active transporter-mediated pathway, accounting for more than half of total hepatic absorption [96]. Members of the organic anion-transporting polypeptides (OATPs) family, which are multi-specific transporters or albumin-binding proteins that bind RVT–albumin complexes and then deliver RVT in a manner similar to fatty acid absorption, are involved in this active process [42].

### 4.3. Metabolism

RVT undergoes substantial phase I (oxidation, reduction, and hydrolysis) and phase II (glucuronic acid, sulfate, and methyl conjugations) biochemical changes in the liver and intestinal epithelial cells shortly after administration, and the resulting metabolites are glucuronic acid and sulfate conjugation [76,97]. The aliphatic double bond is also hydrogenated [98]. While presystemic and systemic conversion to major metabolites (glucuronic and sulfate conjugations) occurs very quickly and efficiently in the intestine and liver in the so-called enterohepatic recirculation, other metabolites such as dihydro-RVT and piceatannol are likely mediated by microbial fermentation of trans-RVT in the gastrointestinal tract. Sulfotransferases (SULTs) sulphation of RVT by sulfotransferases (SULTs) in the human liver yield three metabolites: trans-RVT-3-O-4′-O-disulfate (S1), trans-RVT-4′-O-sulfate (S2), and trans-RVT-3-O-sulfate (S3) [99]. The effects of glucuronidation by uridine 5′-diphospho-glucoronosyl-transferases (UGTs) on RVT intestinal absorption were also examined, and two metabolites, trans-RVT-4′-O-glucuronide (G1) and trans-RVT-3-O-glucuronide (G2), were identified [100].

### 4.4. Excretion

All of RVT’s metabolites are removed from the body pharmacokinetically and excretion is nearly evenly distributed between urine and faeces. RVT and its metabolites are virtually completely eliminated from tissues in 72 h after a single intake. The mono-glucuronides of trans-RVT and dihydro-RVT were the two principal metabolites found in rodent urine [25]. The glucuronide- and sulfate-conjugates of RVT, as well as dihydro-RVT, were the predominant metabolites in humans. The overall recovery of glucuronic and sulphate conjugations in human urine and faeces was approximately 71–98% after oral dosages and 54–91% after intravenous doses, but the aglycone form of RVT had a near-zero recovery [101]. These findings show that the modified metabolite, not the original aglycone, is the most common form of RVT in circulation.

## 5. Safety Profile of RVT

To access its adverse effects, RVT was orally administered at its maximum tolerated levels in multiple toxicity trials. The findings suggest that RVT is not carcinogenic [102]. Furthermore, investigations demonstrated that the compound does not induce acute skin and eye irritation or other allergenicity symptoms [25]. Despite the fact that it is an estrogen-like substance, investigations show that trans-RVT has a low estrogenic potency in vivo. In fact, massive doses of RVT given orally had no effect on reproductive capacity and no substantial changes in bone density. Trans-RVT is found in commercial dietary supplements in amounts ranging from 50 to 500 mg, and human clinical investigations have been conducted up to single dosages of 5 g of the RVT with no adverse effects [103]. These findings indicate that trans-RVT is well tolerated in humans, and that 450 mg of RVT per day is a safe dose for a 70 kg person. A recent epidemiological study found that RVT consumption is inversely connected to breast cancer risk, with 50% or larger decreases in breast cancer risk in women who consumed the most RVT [104]. Given its low toxicity, RVT has been suggested as a possible candidate for chemoprevention in humans. Furthermore, this chemical can cross the blood–brain barrier, implying that it could be used to treat brain illnesses [105]. The summary of clinical trails according to various disorders is shown in Table 4.

## 6. Commercial Products of RVT

Data pertaining to marketed products have been collected from the National Institute of Health, dietary supplement label database and other websites. These items include either RVT or a combination of RVT. These items have a great variation of doses, such as 20 mg per serving to 1400 mg. It indicates a better safety profile of the molecule. An exhaustive list of globally available products has been given in Supplementary Material (Table S2).

## 7. Clinical Trials on RVT Based Products

RVT has also been studied clinically, such as assessment of the effect of RVT on cognitive and cerebral blood flow in the United Kingdom. In Canada, an analysis on the effect of antioxidants on cardio vascular risk was assessed. Furthermore, in Brazil the RVT was examined for its effectiveness in the management of pain due to endometriosis. Moreover, in Taiwan the effect of RVT on complications in patients with haemodialysis was investigated. Additionally, RVT with or without Piperine to enhance the plasma level of RVT was also assessed (USA). Meanwhile, in Singapore, a phase-1 trial is being conducted to analyse the effect of RVT in patients with Type-2 Diabetes (RED). The impact of RVT on brain function and structure was also studied. Moreover, in Italy, RVT’s anti-inflammatory and antioxidants effects on healthy adults were examined. In Tamil Nadu (India), RVT was evaluated as a potent supplement for patient with Type-2 Diabetes Mellitus. Likewise, a similar study was conducted in Maharashtra (India) to confirm whether the addition of RVT is beneficial and safe for patient with diabetes, dyslipidemia and hypertension (who are already on standard therapy). In Karnataka (India), the effect of nutritional supplementation of RVT on patients with advanced cancers and undergoing chemotherapy was evaluated. A similar trial was conducted in Maharashtra (India), to study the effects of nutritional supplementation of extremely active RVT (XAR) in healthy human individuals. In Maharashtra, effect of RVT and copper in reducing toxic side-effects of chemotherapy in patients with advanced mouth cancer was analysed. A similar trial was held in Maharashtra (India), to study the effect of RVT-copper in reducing oral mucositis in patients receiving concurrent chemo radiotherapy for locally advanced oropharyngeal cancer. Additionally, a similar trial was performed in Maharashtra (India) to assess the effect of the addition of RVT with chemotherapeutic agents in patients with gastric cancer. Likewise, the effect of RVT-copper on treating COVID-19 pneumonia was analysed in Maharashtra (India). Additionally, a study to assess the effect of oral RVT and copper combination on life span of glioblastoma patients undergoing surgery was conducted in Maharashtra (India). In Australia, RVT as an option for the treatment of Friedreich ataxia was assessed. A study of dietary RVT on glucose and lipid metabolism disorder is being conducted in China. In Australia, the effect of RVT on circulatory function of obese people with elevated blood pressure was examined. Another trial on similar lines was conducted in Australia where the sustained effect of RVT on circulatory functions in obese adults were assessed. Additionally, the role of RVT in the prevention of colorectal polyps was also investigated in Australia. Moreover, the effect of RVT’s supplementation on gut hormone secretion, gastric emptying and blood glucose responses to meals in patients with type-2 diabetes was assessed in Australia. Meanwhile, another study on RVT supplementation on cerebrovascular function, mood and cognitive performance in type-2 diabetes mellitus (T2DM) was completed in Australia. Another clinical trial was executed in Australia to establish whether RVT can enhance mood, physical function and cerebrovascular function and counteract cognitive decline in post-menopausal women. In Germany, the bioavailability of three different RVT products was evaluated in healthy individuals. Likewise, a similar study was also conducted in Germany, to examine the bioavailability and metabolism of RVT as a constituent of berries in humans. Furthermore, a biokinetic study on the impact of formulation on RVT bioavailability was also performed in Germany. More details about clinical trials are mentioned in Table 5.

## 8. Recent Patent on RVT

The medicinal advantages and other beneficial features of RVT have drawn the attention of numerous researchers to investigate and develop some intellectual property in the form of patents; some of them are included in Table 6.

## 9. Conclusions

This review is an attempt to collectively enlighten readers about the sources, physiological effect, role in human body, pharmacokinetic property, toxicity, commercial products, patents, and clinical trials of RVT. Due to its wide significance as a cancer preventative, cardioprotective, antioxidant, anti-inflammatory, and neuroprotective dietary ingredient, RVT has emerged as one of the most promising naturally occurring compounds with a tremendous therapeutic potential. The preventative and therapeutic efficacy of dietary or supplemental RVT on tumor growth and progression, as well as the prevention of cardiovascular disease and neurological diseases, is being investigated. In fact, RVT and its analogues are pharmacologically safe and can be used with other drugs to improve therapeutic efficacy and reduce toxicity.

Unfortunately, RVT’s pharmacokinetic aspects do not match its positive pharmacological activity. Several investigations have found that trans-RVT is rapidly absorbed, digested, and eliminated in people and animals, implying that RVT has a low bioavailability that undermines its biological effects. The question that arises as to whether RVT can accumulate in target organs to bioactive amounts has yet to be answered. Several research have attempted to answer this topic, but the findings have been mixed. RVT carriers and site-specific delivery methods have been created to protect and stabilise RVT while also increasing its bioavailability and maintaining its biological and pharmacological properties. Despite the advances made in this field, size-tuned carrier systems with optimal lipophilicity are still needed for RVT delivery to more challenging sites such as the brain. Moreover, combination of RVT and conventional chemotherapeutic preparation for the treatment of tumor could be a new option for drug formulation. Due to its wide application, a lot study is required to established its mechanism of action for its pharmacological activities and develop certain delivery methods by which its bioavailability can be improvised without affecting its other properties.

## Figures and Tables

**Table 1:** List of biological sources of resveratrol and their concentration.

S. No	Biological Source	Specified Parts	Concentration of Resveratrol	References
1	Peanut *Arachis hypogaea* Family: Fabaceae	Raw Peanut Kernels	0.02 to 0.31 μg/g	[19,20]
		Peanut leaves,	2.05-μg/g fresh weight	
		Peanut roots,	1.19-μg/g	
		Peanut sprouts,	11.7 to 25.7 μg/g	
		Peanut butter	0.577 to 0.753 μg/g	
2	Grapes *Vitis vinifera*. Family: Vitaceae	Skins of grape berries	24.06 μg/g	[19,20,21,22]
		Red wines	0.352–1.99 μg/mL	
		fruit	3.66 × 10^−2^ (g kg^−1^)	
3	*Ampelopsis cantoniensis* Family: Vitaceae	stem	1.04 × 10^−2^ (g kg^−1^)	
4	Japanese Knotweed Itadori plant (*Polygonum cuspidatum.* Sieb et Zucc) Family: Polygonaceae	Stem	497 µg/g	[19]
		root	523-μg/g	[20]
5	White hellebore (*Veratrum grandiflorum* O. Loes) Family: Melanthiaceae	roots	Not notified	[23]
6	Blueberries *Vaccinium corymbosum* Family: Ericaceae	-	140.0 ± 29.9 pmol/g (or 0.00014 ± 0.00003 μmol/g),	[20]
7	Bilberries *Vacciniu mmyrtillus.* L. Family: Ericaceae	-	71.0 ± 15.0 pmol/g (or 0.000071 ± 0.000015 μmol/g)	[20]
8	Pistachio Nut *Pistaciavera* Family: Anacardiaceae	seeds	0.09–1.67 μg/g	[20]
9	*Paeonia suffruticosaAndr. var. papaveracea* (Andr.) Kerner		8.7 × 10^−1^ g kg^−1^	[21]
10	*Reynoutria japonica* Houtt Polygonaceae	roots	4.209 × 10^−1^ g kg^−1^	[21]

**Table 2:** List of Resveratrol sources with its concentration.

Source	Concentration	References
Wine	0.32–15.35 µg/g	[24]
Peanut butter	0.02–0.98 µg/g	[25]
Peanut	0.01–0.07 µg/g	[26]
Green peanuts	0.19–0.72 µg/g	[24]
Polygonum cuspidatum	296–377 µg/g	[16]
Green grapes	0.02–0.32 µg/g	[27]
Black graps	0.95–1.88 µg/g	[24]
Risins	0.0005–0.003 µg/g	[28]
Grape juice-black	Traces-0.09 µg/g	[29]
Grape juice-green	Traces-0.01 µg/g	[30]
White wines (Spanish)	0.05–1.80 mg/L	[31]
Rose wine (Spanish)	0.43–3.52 mg/L	[32]
Red wine (Spanish)	1.92–12.59 mg/L	[33]
Red wine (global)	1.98–7.13 mg/L	[34]
Red grape juice (Spanish)	1.14–8.69 mg/lL	[35]

**Table 3:** Mechanism of action of Resveratrol for different conditions.

S. No	Therapeutic Effect	Mechanism of Action	References
1.	Antioxidant activity	Several antioxidant enzymes, including glutathione peroxidase, glutathione S-transferase, and glutathione reductase, were enhanced by resveratrol. Resveratrol also has antioxidant capability for the preservation of polyunsaturated fatty acids (PUFA).	[44,45]
2.	Platelet Aggregation	Platelet membrane-bound fibrinogen and protein kinase C inhibitor (PKCI) demonstrated an additive impact in decreasing platelet aggregation content.	[46]
3.	Enzyme Inhibitors	In the animal cell system, trans-resveratrol inhibits oxidative enzymes. Superoxide dismutase, lipoxygenase, catalase, peroxidase, polyphenol oxidase, and 1-aminocyclopropane-1-carboxylic acid oxidase were all inhibited. Trans-resveratrol inhibits lipoxygenase activity more effectively than other lipoxygenase inhibitors such propyl gallate, ibuprofen, ursolic acid, acetylsalicylic acid, and salicyl-hydroxamic acid (Fan and Matthesis, 2001).	[47,48]
4.	Anti-carcinogenic Agents	Resveratrol’s anti-initiation effect has been connected to the inhibition of metabolic activation and/or promotion of carcinogen detoxification via manipulation of enzymes engaged in either phase I (i.e., cytochrome P450 enzymes (CYP)) or phase II conjugation processes. Several in vitro investigations have revealed that resveratrol suppresses the activity of the CYP1A1 and CYP1A2 enzymes.	[49]
5.	Anti-neoplastic and phytogenic agents	Gastric adenocarcinoma cells respond to resveratrol therapy by suppressing DNA synthesis, activating nitric oxide synthase, inducing apoptosis, and inhibiting total PKC and PKC alpha activity.	[24]
6.	Arthritis	The transcription factor nuclear factor-kappa B is activated, which regulates all of these factors. Thus, any medication that inhibits the production of tumour necrosis factor-alpha, interleukin-1beta, cyclooxygenase-2, lipoxygenase, matrix metalloproteinases, or adhesion molecules, or inhibits NF-kappa B activation, has the potential to treat arthritis.	[50]
7.	Cardiovascular Diseases	Resveratrol may protect the cardiovascular system against ischemic-reperfusion damage, as well as protect and preserve the endothelium. It also has anti-atherosclerotic characteristics, reduces LDL oxidation, suppresses platelet aggregation, and has estrogen-like activities.	[51,52]
8.	Diabetes	By activating the NAD (+)-dependent histone deacetylase Sirt1, resveratrol improved life duration in lower species. Resveratrol was also discovered to boost lifespan and glucose homeostasis in mice via activating Sirt1-mediated deacetylation of the transcriptional coactivator PGC-1alpha.	[53,54]
9.	Anti-inflammatory	Resveratrol has anti-inflammatory effects through modulating enzymes and pathways that create inflammatory mediators, as well as inducing programmed cell death in activated immune cells.	[52]

**Table 4:** Summary of Resveratrol’s clinical effects.

Disease Type	Study Conditions	Length of Trial	Resveratrol Dosage	Biomarker Changes	Effect	References
**Anti-canc** **er**						
Prostate cancer	14 patients, phase 1 trial	2–31 months (depending on patient)	500, 1000, 2000, 3000, or 4000 mg of MPX. Every 500 mg MPX has 4.4 μg resveratrol	IncreaseinPSADT	Beneficial	[106]
Prostate cancer	66 patients, randomized, placebo-controlled, single-site clinical trial	4 months	150 mg or 1000 mg daily	Decrease in androstenedione, DHEA, and DHEAS. No effect on prostate size and PSA levels	None	[107]
Colorectal cancer	9 patients randomized, placebo- controlled, double blind, phase 1 trial	14 days prior to surgery	5.0 g SRT501	Increase in cleaved Caspase-3 (apoptosis)	Beneficial	[108]
Colorectal cancer	20 patients	8 days priortosurgery	500 or 1000 mg	Reduction in tumor cell proliferation, indicated by reduction in Ki-67 staining	Beneficial	[109]
Multiple-myeloma	24 patients, phase 2 trial	~4 months	5.0 g SRT501	NA	Severe adverse events	[110]
Breast cancer	39 patients r andomized, double-blind, placebo-controlled clinical trial	3 months	5 or 50 mg twice daily	Decrease in *RASSF-1α* methylation	Beneficial	[111]
**Neurological disorders**						
Alzheimer disease	119 patients, randomized, placebo-controlled, doubleblind, multi-site, phase 2 trial	12 months	500 mg once daily, with 500 mg dose escalation every 13 weeks, ending with 1000 mg twice daily	Reduced CSF MMP9, increase IL-4, attenuated decline in Aβ42 and Aβ40	Beneficial	[112]
Alzheimer disease	119 patients, randomized, placebo-controlled, double-blind, multicenter, phase 2 trial	12 months	500 mg once daily, with 500 mg dose escalation every 13 weeks, ending with 1000 mg twice daily	Attenuated decline in Aβ42 and Aβ40 increased brain volume loss	Beneficial	[112]
Ischemic stroke	312 patients, randomized, placebo-controlled	60 min after 0–2 h of stroke onset	2.5 mg resveratrol/kg ofbodyweight	Reduced MMP-9 and MMP-2	Beneficial	[113]
**Cardiovascular diseases**						
Coronary artery disease	40 patients, double-blind, randomized, placebo-controlled	3 months	10 mg daily	Improved left ventricular systolic and diastolic function; improved FMD; lowered LDL-cholesterol level	Beneficial	[114]
Atherosclerosis	44 healthy subjects, double-blind randomized, placebo-controlled	1 month	400 mg trans-resveratrol, 400 mg grapeskin extract, 100 mg quercetin	Decreased expression of endothelial cell ICAM, VCAM and IL-8; decreased levels of plasma IFN-γ and insulin	Beneficial	[114]
Hypertension	18 patients, double-blind, randomized, placebo-controlled, crossover design	28 days	330 mg grape seed and skin, 100 mg green tea, 60 mg resveratrol, 60 mg blend of quercetin, ginkgo biloba and bilberry	Reduced diastolic pressure	Beneficial	[115]
Inflammation and oxidative stress	50 healthy adult smokers, double- blind, randomized, crossover design	3 month	500 mg daily	Reduced systemic inflammation in airways, decreased CRP release from the liver	Beneficial	[116]
Serum glucose and cardiovascular risk factor	19 schizophrenic male patients, double-blind, randomized, controlled	1 month	200 mg daily	No change in body weight, waist circumference, glucose, and total cholesterol	None	[117]
Cardiovascular health of overweight and obese subject	45 overweight and slightly obese subjects, randomized, placebo- controlled, crossover design	1 month	150 mg daily	No change in apoA-I concentrations and HDL level	None	[118]
**Diabetes**						
Type 2 diabetes, hyperglycaemia	62 patients, prospective, open-label, randomized, controlled trial	3 months	250 mg daily	Improved glycemic control: decreased HbA1c, systolic BP, total cholesterol, and total protein	Beneficial	[119]
IGT	10 patients with mean age 72 ± 3 years, open-label study	1 month	1000, 1500, or 2000 mg daily	Decrease in peak postmeal glucose and 3-h glucose, increased insulin sensitivity	Beneficial	[120]
**NAFLD (Non-alcoholic fatty liver disease)**						
NAFLD	28 patients, randomized, placebo- controlled	6 months	1500 mg daily	No change in ALT No improvement in lipid profile or insulin sensitivity	None	[121]
NAFLD	60 patients, randomized, placebo-controlled, double blind	3 months	300 mg twice daily	Reduced AST, ALT, cholesterol, glucose, TNF-o	Beneficial	[122]
NAFLD	50 patients, randomized, double-blind, placebo-controlled	3 months	500 mg (in addition to exercise and healthy diet)	Reduction in ALT, IL-6, NF- B activity improved lipid profiles	Beneficial	[123]

**Table 5:** List of global clinical trials on Resveratrol.

S. No.	CT Number	Title of the Study	Status/Phase	Condition	Sample Size	Sponsor	Location/Country
1.	NCT01010009	The Cognitive and Cerebral Blood Flow Effects of Resveratrol	Completed PHASE: Not Applicable	Cognitive and Cerebral Blood Flow Effects of Resveratrol	24 participants	North Umbria University	North Umbria University Newcastle upon Tyne, United Kingdom
2.	NCT01964846	Effect of Antioxidant Intake on Cardiovascular Risk	Completed PHASE: Not Applicable	Effect of Resveratrol and Curcumin on Inflammation	22 participants	Laval University Atrium Innovations	Institute on Nutrition and Functional Foods (INAF), Laval University Québec City, Quebec, Canada
3.	NCT02475564	Resveratrol for Pain Due to Endometriosis (ResvEndo)	Completed Phase 4	Endometriosis	44 participants	Hospital de Clinicas de Porto Alegre	HCPA Porto Alegre, RS, Brazil
4.	NCT03446625	Resveratrol as a Preventive Treatment of OHSS (RES-OHSS)	Completed Phase 3	Infertility	70 participants	IVI Madrid	Ivi Madrid Madrid, Spain
5.	NCT03352895	The Effects of Resveratrol on the Complications of Patients With Hemodialysis	Completed PHASE: Not Applicable	Chronic Kidney Disease	36 participants	Dalin Tzu Chi General Hospital	Dalin Tzu Chi Hospital Chiayi City, Taiwan
6.	NCT01324089	Resveratrol With or Without Piperine to Enhance Plasma Levels of Resveratrol	Completed Phase: Not Applicable	Focus of the Study: Normal Volunteers	24 participants	University of Wisconsin, Madison National Institutes of Health (NIH) National Cancer Institute (NCI)	University of Wisconsin Madison, Wisconsin, United States
7.	NCT01677611	Effects of Resveratrol in Patients With Type 2 Diabetes (RED)	Completed Phase 1	Type 2 Diabetes	10 participants	Khoo Teck Puat Hospital National Medical Research Council (NMRC), Singapore	Alexandra Health, Khoo Teck Puat Hospital Singapore, Singapore
8.	NCT02433925	Resveratrol’s Effects on Inflammation and Oxidative Stress in Chronic Kidney Disease	Completed Phase 3	Chronic Renal Insufficiency	20 participants	Universidade Federal Fluminense	No Contacts or Locations Provided
9.	NCT02621554	Impact of Resveratrol on Brain Function and Structure	Completed Phase: Not Applicable	Healthy	60 participants	Max Planck Institute for Human Cognitive and Brain Sciences University of Leipzig Evolva SA	Department of Neurology, Max Planck Institute for Human Cognitive and Brain Sciences Leipzig, Germany
10.	NCT01492114	Anti-inflammatory and Antioxidant Effects of Resveratrol on Healthy Adults	Completed Phase 3	Chronic Subclinic Inflammation Redox Status	40 participants	University of Turin, Italy	Simona Bo Turin, Italy
11.	CTRI/2011/05/001731	Evaluation of Resveratrol as a Dietary Supplement in Type 2 Diabetes Mellitus	Applicable only for Completed/Terminated trials Phase 4	Type 2 Diabetes Mellitus	60 participants	Dr MJ Nanjan JSS College of Pharmacy, (Off Campus of JSS University, Mysore) Post Box 20, Rocklands, Ootacamund-643001 The Nilgiris, Tamilnadu Research institution	Outpatient Department of Government headquarter hospital, Ootacamund, Tamil Nadu
12.	CTRI/2017/04/008384	A study to check whether addition of Resveratrol is beneficial and safe in patients with Diabetes, Dyslipidemia and Hypertension (who are already on standard therapy)	Phase 4 Completion date missing	Dyslipidemia, Diabetes and hypertension	180 participants	Rakesh Ojha Dept of Pharmacology, Uka Tarsadia University, Gopal Vidyanagar, Bardoli Mahuva Road,, Tarsadi, Bardoli, Surat, Pin code: 394350	Department of Medicine, Vikas Hospital, Maharashtra
13.	CTRI/2017/04/008376	To study the effects of nutritional supplementation of resveratrol on fatigue and quality of life in patients with advanced cancers undergoing chemotherapy.	Applicable only for Completed/Terminated trials N/A	Patients diagnosed with advanced cancers undergoing non-biological, second-line chemotherapy.	40 participants	Dr Vijay Agarwal HCG Towers, No. 8, P. Kalinga Rao Road, Sampangi Ram Nagar, Bangalore-560027	HealthCare Global Specialty Hospital, Karnataka
14.	CTRI/2017/09/009694	To study the effects of nutritional supplementation of extremely active resveratrol (XAR) in healthy human individuals.	Applicable only for Completed/Terminated trials N/A	Healthy human volunteers not suffering from any disease	100 participants	Epigeneres Biotech Pvt Ltd. A-206, Dalamal Towers, Free Press Journal Marg, Nariman Point, Mumbai-400021	National facility for Biopharmaceutical, Maharashtra
15.	CTRI/2018/03/012459	A study to assess the effect of Resveratrol and Copper in reducing toxic side-effects of chemotherapy in patients with advanced mouth cancer.	Applicable only for Completed/Terminated trials N/A	Operable stage IV squamous cell carcinoma of buccal mucosa patients planned for surgery who have not received any prior treatment.	25 participants	TMC Research Administrative Council Department of Atomic Energy, Government of India; Tata Memorial Centre; Parel. Mumbai. India. PIN:400011 Government funding agency	Tata Memorial Hospital, Maharashtra
16.	CTRI/2019/06/019500	To study the effect of Resveratrol-Copper in reducing oral mucositis in patients receiving concurrent chemo-radiotherapy for locally advanced oropharyngeal cancer.	Applicable only for Completed/Terminated trials Phase 2	Malignant neoplasm of oropharynx, unspecified	102 participants	Tata Memorial Hospital Dr. E Borges Road, Parel, Mumbai, Maharashtra 400012 Research institution and hospital	Tata Memorial Hospital, Mumbai, Maharashtra
17.	CTRI/2019/07/020289	Addition of resveratrol copper with chemotherapy in gastric cancer patients	Applicable only for Completed/Terminated trials Phase 2	Malignant neoplasm of stomach, unspecified	42 participants	Tata Memorial Hospital Dr Ernest Borges Marg Parel Mumbai Research institution and hospital	Tata Memorial Hospital, Maharashtra
18.	CTRI/2020/06/026256	Resveratrol and copper for the treatment of COVID-19 pnuemonia.	Applicable only for Completed/Terminated trials N/A	Coronavirus as the cause of diseases classified elsewhere	230 participants	TopiwalaNational Medical College and BYL Nair charitable Hospital Dr Anandrao Nair Marg, Mumbai Central, Mumbai, Maharashtra 400008 Government Medical college	T.N.M.C and B.Y.L.Nair Hospital, Maharashtra
19.	CTRI/2020/09/027794	Role of Copper in addition to whole brain radiotherapy in brain metastases in lung cancer	Applicable only for Completed/Terminated trials Phase 2	Malignant neoplasm of unspecifiedpart of bronchus or lung	120 participants	Jai Prakash Agarwal Room No 1131, Homi Bhabha Block, Department of Radiation Oncology, Tata Memorial Hospital, Parel, Mumbai	Tata Memorial Hospital, Maharashtra
20.	CTRI/2020/10/028476	Study to assess the effect of oral resveratrol and copper combination on life span of glioblastoma patients undergoing surgery.	Applicable only for Completed/Terminated trials Phase 1/ Phase 2	Other specified disorders of brain	66 participants	Tata Memorial Centre TMC Tata Memorial Hospital Dr E Borges Road Parel Mumbai Research institution and hospital	Tata Memorial Hospital, Maharashtra
21	NCT01339884	A Study of Resveratrol as Treatment for Friedreich Ataxia	Completed Phase 1 Phase 2	Friedreich Ataxia	27 participants	Murdoch Children’s Research Institute Friedreich’s Ataxia Research Alliance	Monash Medical Centre, Southern Health Clayton, Melbourne, Victoria, Australia
22.	ChiCTR2100043997	A dose-response study of dietary resveratrol on glucose and lipid metabolism disorder	Recruiting	glucose and lipid metabolism disorder	40 participants	Department of Nutrition, School of Public Health, Sun Yat-Sen University	Huanghuagang Street Community Health Service Center, Yuexiu District, Guangzhou City, China
23.	ACTRN12609000023257	Acute effects of resveratrol on circulatory function in obese people with elevated blood pressure.	Completed Phase Not Applicable	Endothelial vasodilator function Blood pressure response to exercise	20 participants	Professor Peter Howe Nutritional Physiology Research Centre University of South Australia, PO Box 2471 Adelaide, South Australia, 5001 Australia	Nutritional Physiology Research Centre University of South Australia, PO Box 2471 Adelaide, South Australia, 5001
24.	ACTRN12611000060943	Sustained effects of resveratrol on circulatory function in obese adults	Completed Phase Not Applicable	Endothelial vasodilator function Cognitive function	30 participants	Professor Peter Howe Nutritional Physiology Research Centre, University of South Australia PO Box 2471 Adelaide, 5001 Australia Dr Narelle Berry University of South Australia PO Box 2471 Adelaide, 5001 Australia	Clinical Nutrition Research Centre University of Newcastle School of Biomedical Sciences & Pharmacy Callaghan NSW 2308 Australia
25.	ACTRN12611000560998	Resveratrol in the prevention of colorectal polyps	Completed Phase Not Applicable	high risk/familial risk of colorectal cancer	128 participants	Melbourne Health Royal Melbourne Hospital City Campus, Grattan St, Parkville. Vic 3050 Australia Australian Wine Research Insititute PO Box 197, Glen Osmond SA 5064 Australia	Level 3 Centre, Department of Colorectal Medicine & Genetics Royal Melbourne Hosptial Parkville, VIC, 3050 Australia
26.	ACTRN12611001288910	The effect of resveratrol in red wine on cognitive function in older adults: Preliminary study	Completed Phase Phase 1/Phase 2	Cognitive function in older adults Absorption rates of resveratrol	2 participants	Swinburne University of Technology PO Box 218 Hawthorn VIC 3122 Australia	H24, Po Box 218 Hawthorn, Vic, 3122 Australia
27.	ACTRN12613000717752	The effect of resveratrol supplementation on gut hormone secretion, gastric emptying, and blood glucose responses to meals in patients with type 2 diabetes	Completed Phase Phase 2	type 2 diabetes mellitus	15 participants	Royal Adelaide Hospital North Terrace Adelaide South Australia 5000 Australia	Discipline of Medicine Royal Adelaide Hospital North Terrace Adelaide SA 5000 Australia
28.	ACTRN12615000291583	Assisting post-menopausal women towards healthy aging-can resveratrol enhance mood, physical function and cerebrovascular function and counteract cognitive decline?	Completed Phase Phase 3	Menopause Cognitive function	80 participants	University of Newcastle University Drive Callaghan NSW 2308 Australia	Clinical Nutrition Research Centre School of Biomedical Sciences & Pharmacy Medical Sciences Building, MS 514 University of Newcastle University Drive Callaghan NSW 2308 Australia
29.	DRKS00021683	Bioavailability of three different Resveratrol products in healthy subjects—a randomized, double-blind three-way cross-over study	complete Phase: N/A	Resveratrol Bioavailability	15 participants	Wacker Chemie AG Ms. Rachela Mohr Hanns-Seidel-Platz 4 81737 München Germany	Biotesys GmbH, Esslingen am Neckar
30.	DRKS00004311	Biokinetic studies on the impact of formulation on resveratrol bioavailability	complete Phase: N/A	metabolism of resveratrol in healthy volunteers	12 participants	Max Rubner-Institut Haid-und-Neu-Str. 9 76131 Karlsruhe Germany	Max Rubner-Institut, Karlsruhe
31.	DRKS00008788	Investigation on the bioavailability and metabolism of resveratrol an ingredient of berries in humans	complete Phase: N/A	healthy volunteers	100 participants	Max Rubner-InstitutBundesforschungsinstitut für Ernährung und Lebensmittel Haid-und-Neu-Str.9 76131 Karlsruhe Germany	Max Rubner-Institut; Bundesforschungsinstitut für Ernährung und Lebensmittel, Karlsruhe

**Table 6:** List of Patent on Resveratrol Globally.

S. No.	Patent Publication Number	Country	Title of the Patent	Details of the Invention
1.	US20160215306A1	USA	Method for Producing Modified Resveratrol	This Invention Generates Glycosylated and Methylated Resveratrol in a Genetically Modified Cell, Through Two Methods, Bioconversion And In Vitro.
2.	CN108815116	China	Resveratrol Ointment Capable of Curing Pathological Scars, Preparation Method and Application	The Invention Reveals Resveratrol Ointment Which Is Capable of Curing Pathological Scars, A Preparation Method and Application.
3.	EP2007366	Europe	Animal Product Enrichment Using Resveratrol	Combining Resveratrol with a Bioavailability Promoter to Make a Bioavailable Resveratrol is one of the methods and systems disclosed herein.
4.	US11103465B2	USA	Trans-Resveratrol Topical Medication for The Treatment of Pain and Method of Manufacture Thereof	A Topical Pain Medication and Method of Their Manufacture. In one Embodiment, The Medication Includes: (1) Enriched Resveratrol Having a Trans Concentration of Resveratrol More Than That of Naturally Occurring Trans-Resveratrol (2) At Least One Inactive Ingredient Configured to Mix with The Resveratrol to Form the Topical Medication.
5.	CN108354915	China	Medicine For Treating Cervical Cancer by Formula of Combined Resveratrol and Sodium Pyruvate, And Preparation Method	The Invention Reveals a Medicine for Treating Cervical Cancer by A Formula of Combined Resveratrol and Sodium Pyruvate.
6.	CN109602702	China	Resveratrol Nanoparticles with Brain Targeting Function and Preparation Process Thereof.	A Resveratrol Nanoparticle Has Brain Targeting Function. According To Preparation Provided by The Invention, The Lactoferrin Modified Resveratrol Nanoparticles, Which Enhances the Brain-Targeting and Has an Anti-Ad Effect.
7.	CN108042515	China	Application of Resveratrol in Preparation of Estrogen Level Adjusting Medicines and Pharmaceutic Preparation of Resveratrol	The Invention Discloses Application of Resveratrol in Preparation of Estrogen Level Adjusting Medicines and A Pharmaceutic Preparation of The Resveratrol.
8.	CN110339170	China	Resveratrol Nanocomposite Powder and Preparation Method Thereof	The Invention Discloses Resveratrol Nanocomposite Powder and A Preparation Method.
9.	CN103550161	China	Resveratrol Loaded Polyurethane Micro-Nano Particle and Preparation Method Thereof	The Invention Relates to Resveratrol Loaded Polyurethane Micro-Nano Particles and A Preparation Method Thereof.
10.	CN107951870	China	Medicinal Composition Containing Resveratrol and Being Capable of Inhibiting Drug-Resisting Staphylococcus Aureus	The Invention Belongs to The Field of Pharmacy, And Relates to Compound 3, 4′,5-Trihydroxy-Diphenyl (Resveratrol) And Applications of The Compound 3, 4′,5-Trihydroxy-Diphenyl (Resveratrol) In Preparing Sensitivity Enhancing Medicines of Methicillin-Resistant Staphylococcus Aureus Capable of Resisting Fluoroquinolones.
11.	CN102276667	China	High-Efficiency Anti-Liver Cancer Resveratrol Prodrug and Synthetic Method Thereof	The Invention Discloses High-Efficiency Anti-Liver Cancer Resveratrol Prodrug and Synthetic Method Thereof.
12.	CN107213142	China	Application Of Oxidized Resveratrol and Combined Oxidized Resveratrol and Antibiotic in Preparation of Anti-Fungal-Infection Product	The Invention Relates to Application of Oxidized Resveratrol and Combined Oxidized Resveratrol and Antibiotic in The Preparation of An Anti-Fungal-Infection Product, Belonging to The Technical Field of Medicines.
13.	CN106176772	China	Health care food containing resveratrol and preparing method of health-care food	The Invention Relates to A Health-Care Food Containing Resveratrol and A Preparing Method of The Health-Care Food.
14.	CN101371827	China	Resveratrol Composition for Delaying Age	The Invention Relates to An Anti-Aging Combination of Resveratrol. The Technical Proposal of The Invention Is That the Anti-Aging Combination Consists of Resveratrol and Quercetin, And the Compatibility Proportion Is 40–350 mg Of Resveratrol And 8–200 mg Of Quercetin.
15.	CN107281107	China	Polyethylene Glycol Modified Resveratrol Magnetic Nanoliposomes and Preparation Method Thereof	The Invention Discloses Polyethylene Glycol Modified Resveratrol Magnetic Nanoliposomes Covered with Ferro Ferric Oxide.
16.	CN110041173	China	Novel Resveratrol as well as Derivative Synthesis Method and Application Thereof	The Invention Belongs to The Technical Field of Organic Synthesis and Discloses Novel Resveratrol as Well as A Derivative Synthesis Method and Application Thereof.
17.	CN111423444	China	Resveratrol-Temozolomide Eutectic Crystal and Preparation Method and Application Thereof	The Invention Aims to Provide Resveratrol-Temozolomide Eutectic Crystal, that can improve the Solubility of Medicine, Improve the Stability and Improve the Bioavailability, and Also Provides a Preparation Method and Application of The Resveratrol-Temozolomide Eutectic Crystal.
18.	WO2006000603	Spain	Use Of Trans-Resveratrol as Therapeutic Agent for the Treatment of Male Infertility and/or Subfertility in Mammals	The Invention Relates to The Use of Trans-Resveratrol as A Therapeutic Agent for The Treatment of Male Infertility and/or Subfertility in Mammals.
19.	EP1076556	Europe	Administration Of Resveratrol to Prevent or Treat Restenosis Following Coronary Intervention	A Method for Preventing or Treating Restenosis and For Preventing the Recurrence or Progression of Coronary Heart Disease Is Provided.
20.	JP2016088858	Japan	Resveratrol Derivative that Generates Hydrogen and Exhibits Keratin Producing Action and Method for Producing the Derivative	To provide a Resveratrol Derivative that Generates Hydrogen and Exhibits Keratin Producing Action, which is Useful as A Cosmetic, Food Preparation, Medicine, And, Especially When used in Cosmetic, Expected to Improve Degradation of Sebum, Moisture Holding Power of Skin, And Resilience of Skin.
21.	EP3082780	Europe	Combination of Bezafibrate and of Resveratrol or Resveratrol Derivatives for Treatment and Prevention of diseases involving a Mitochondrial energy dysfunction	The Present Invention Relates to the combined use of bezafibrate and of Resveratrol and its derivatives for the treatment of diseases involving mitochondrial energy dysfunction, and also to a pharmaceutical kit comprising both Bezafibrate and Resveratrol and its derivatives.
22.	ES2685094T3	Spain	Cosmetics composition containing Resveratrol	Resveratrol, A Component of a Variety of Common Edible Plants, Including Peanuts and Red Grapes, Is A Phytoestrogen.
23.	CN101579291	China	Resveratrol Phospholipid Composite Nano-Emulsion and Preparation Method and Application Thereof	The Invention discloses Resveratrol Phospholipid composite Nano-Emulsion and preparation method and application Thereof.
24.	EP1138323	Europe	Resveratrol For the Treatment of Exfoliative Eczema, Acne or Psoriasis	The Use of Resveratrol (3,4′,5-Trihydroxy-Trans-Stilbene) and Derivatives Thereof, for the Preparation of Medicaments for the Treatment of Exfoliative Eczema, Acne and Psoriasis, Topical Pharmaceutical Formulations Containing Resveratrol or Derivatives Thereof in Combination with Other active principles.
25.	CN102000045	China	Application Of Resveratrol in Preparing Medicament for Preventing and Treating Radiation Induced Depigmentation Skin Disease	The Invention Discloses Application of Resveratrol in Preparing a Medicament for Preventing and Treating Radiation Induced Depigmentation Skin Disease.
26.	KR1020080012483	South korea	Transgenic Rice Containing Resveratrol Having Medicinal Effects on Cancer, Hyperlipidemia and Thrombosis	Rice Containing Resveratrol Having Anticancer, Anti-Hyperlipidemia and Antithrombic Effects in Rice by Using a Gene Encoding Resveratrol, so that the Rice is useful for Production of Various Processed Products including Food, Cosmetics, Drink and Fodder.
27.	WO2015126129	Korea	Resveratrol Multimer Having Selective Inhibitory Activity for Hepatitis C Virus Genome Replication, and Use Thereof	Invention Concerns Resveratrol Multimer with Selective Inhibitory Activity for Hepatitis C Virus Genome Replication and Its Use, And More Particularly a Pharmaceutical Composition for Preventing or Treating Hepatitis C, Containing the Same.
28.	CN110812360	China	Application Of Resveratrol and Combination of Resveratrol and Ketoconazole in Preparation of Medicaments for Resisting Fungal Infection Diseases	The Invention Discloses an Application of Resveratrol and Combination of Resveratrol and Ketoconazole in Preparation of Medicaments for Resisting Fungal Infection Diseases.
29.	EP2249806	Europe	Resveratrol Formulations	Resveratrol Can Be Used in Large Quantity to Treat or Inhibit the Onset of Many Diseases that Are Related to The Aging Process. the Present Invention Provides Concentrated Liquid Formulations of Resveratrol, typically having a Resveratrol Concentration of at least 10% By Weight.
30.	WO2011097691	Brazil	Composition containing resveratrol and its Derivatives Thereof and Plant Oil, Process for Producing Said Composition, Nutraceutical and/or Pharmaceutical Product, and Method for Enhancing the Potential of Resveratrol	Chemical has been developed to produce substances that have anti-inflammatory, antiviral, cardiovascular, neuroprotective and cancer-preventive properties
31.	US6572882B1	USA	Compositions based on resveratrol	Cancer Chemo preventive Activity of Resveratrol, a Natural Product Derived from Grapes
32.	US6414037B1	USA	Pharmaceutical formulations of resveratrol and methods of use thereof	Historical context, evaluation of the transformation assay, and evolution and optimization of the transformation assay methodology in C3H10T1/2 C18 mouse embryo fibroblasts.
33.	US10780056	USA	Resveratrol delivery system	The potential of resveratrol as a treatment for neurodegenerative and other diseases must be thoroughly investigated.
34.	US8642660	USA	The influential patent that’s driving anti-aging research	It could one day lead to treatment for parkinson’s and alzheimer’s slow down aging or even extended life expectancy.

## Data Availability

Data sharing not applicable.

## Supplementary Materials

The following supporting information can be downloaded at: https://www.mdpi.com/article/10.3390/ph15080957/s1.
