# Supplementary material for: Resveratrol from Dietary Supplement to a Drug Candidate: An Assessment of Potential
Source: Pharmaceuticals (Basel). 2022 Aug 1;15(8):957. doi: 10.3390/ph15080957 (PMC9412308; doi:10.3390/ph15080957)
Supplement: Supplementary file 1 [file pharmaceuticals-15-00957-s001.zip › pharmaceuticals-1826911-supplementary.pdf]

Table S1: Physico-chemical properties of Resveratrol

Property	Values
Relative density	1.359 g/cm^3
Referactive index	1.762
Solubility	0.05 mg/mL in water to 374 mg/mL in polyethylene glycol 400 (PEG-400)
Vapor pressure	0.0±1.1 mmHg at 25°C
Octanol/ water partition coefficient	2.25±0.085/ 3.32
Flash point	222.3±14.7 °C
Melting point	261 to 263 °C (502 to 505 °F; 534 to 536 K)
Boiling point	449.1±14.0 °C at 760 mmHg

Table S2: List of dietary supplement / botanicals / nutraceutical products available in global market.

S.No	Brand Name	Pack size/Net Contents	Serving Size	Amount of Resveratrol/serving unit	Manufacturer/Distributor	Website
1.	21st Century Resveratrol red wine extract capsules	90 Capsules	1 Capsule(s)	200 mg	21st century healthcare Inc. USA	https://www.21stcenturyvitamins.com/products/specialty/other-specialty/resveratrol-red-wine-extract
2.	Beautiful Ally by Bluebonnet	30 Vegetable Capsules	1 Capsule(s)	500 mg	Bluebonnet Nutrition Corp.	https://bluebonnetnutrition.com/product/beautiful-ally-resveratrol-500-mg-30-vegetable-capsules/
3.	BioGenesis Nutra BioGenesis Resveratrol Plus Flavonoids	90 Veggie Capsules	3 Capsule(s)	250 mg	Nutra BioGenesis	https://kiwla.com/products/biogenesis-resveratrol-plus-flavonoids
4.	Biovea Resveratrol	60 Vegetarian Tablet(s)	1 Vegetarian Tablet(s)	250 mg	Biovea,USA	https://www.biovea.com/eu/product/detail/1967/resveratrol-250mg-60-vegetarian-tablets
5.	Biovea resveratrol	60 Vegetarian Capsule(s)	1 Vegetarian Capsule(s)	40 mg	Biovea,USA	https://www.biovea.com/eu/product/detail/2044/resveratrol-40mg
6.	Botanic Choice Resveratrol	30 Capsules	1 Capsule(s)	100 mg	Botanic Choice	https://www.botanicchoice.com/Resveratrol/Resveratrol-100-mg-30-capsules.axd
7.	Botanic Choice Resveratrol Plus™	30 Capsules	1 Capsule(s)	150 mg	Botanic Choice	https://www.botanicchoice.com/Resveratrol/Resveratrol-Plus-30-capsules.axd

**8.**	California Academy of Health Resveratrol Natur'elle	60 Capsule(s)	2 Vegicap(s)	100 mg	Made in the U.S.A.	https://kusoglife.com/products/california-academy-of-health-resveratrol-naturelle/
**9.**	CurEase<br>100% Tran Resveratrol Powder	2.1 Oz<br>240 SERVINGS<br>60 g	0.125 Teaspoon(s)	250 mg	CurEase	https://www.curease.com/
**10.**	CurEase Trans Resveratrol 1600 with Acai Berry Antioxidant	60 Veggie Capsule(s)	2 Capsule(s)	1000mg	CurEase	https://www.curease.com/
**11.**	CurEase TransResveratrol Grape Skin Acai 60,000 mg Powder	60000 Mg<br>120 servings	0.25 Teaspoon(s)	500 mg	CurEase	https://kusoglife.com/products/curease-trans-resveratrol-grape-skin-acai-60-000-mg-powder/
**12.**	CurEase ResveraDog Resveratrol Anti-Aging Formula Powder for Dogs	60 Servings	2 scoops per Serving	50 mg	CurEase	https://www.curease.com/
**13.**	Doctor's Best High Potency Trans-Resveratrol	60 Veggie Capsules	1 Veggie Capsule(s)	600 mg	Doctor's Best. Inc California, USA	https://www.drbvitamins.com/shop/trans-resveratrol-600mg-60-veggiecaps
**14.**	Douglas Laboratories Methylated Resveratrol Plus	30 Vegetarian Capsules	1 Vegetarian Capsule(s)	100 mg	Douglas Laboratories Pittsburgh,USA	https://www.nhc.com/methylated-resveratrol-plus-by-douglas-laboratories
**15.**	Dr. Sinatra Omega Q Plus,	60 Softgels	2 Softgel(s)	30 mg	Sinatra	https://in.iherb.com/pr/dr-sinatra-omega-q-

	Resveratrol Turmeric					plus-resveratrol-turmeric-60-softgels/101159
**16.**	Earth Wise Vitamins & Supplements Resveratrol Plus	60 Capsule(s)	1 Capsule(s)	400 mg	Earth Wise	https://kusoglife.com/products/earth-wise-vitamins-and-supplements-resveratrol-plus/
**17.**	Endurance Products Company Resveratrol	150 Tablets	1 Tablet(s)	100mg	Endurance Products Company	endur.com/products/resveratrol-dietary-supplement-150-tablets-100mg
**18.**	Gaia Herbs Professional Solutions Resveratrol 150	50 Liquid-Filled Capsules	2 Capsule(s)	150 mg	Gaia Herbs Inc.	https://www.gaiaherbs.com/products/resveratrol-150
**19.**	Genceutic Naturals Wild Crafted Resveratrol 100mg	60 Vegetarian Capsules	1 Capsule(s)	100mg	Nature's Answer,USA	https://genceutic.com/product/wild-crafted-resveratrol-100mg/
**20.**	Genestra Brands Resveratrol 250	60 Vegetable Capsules	1 Capsule(s)	250 mg	Seroyal USA	https://www.chiroeco.com/products/product/resveratrol-250/
**21.**	HerbaSway Resveratrol – Anti-Aging Support	2 fl. Oz.	1 mL	-	HerbaSway Laboratory LLC	https://www.herbasway.com/product/resveratrol-anti-aging-support/
**22.**	Hi-Tech Pharmaceuticals resveratrol	90 Tablets	1 Tablet(s)	500 mg	Hi-Tech Pharmaceuticals	https://www.mysupplementstore.com/products/htp-resveratrol-500mg-90t
**23.**	High land Laboratories Red Wine Resveratrol Tablet	90 Lozenges	1 Vegetarian Lozenge(s)	25 mg	Highland Labs	highlandvitamins.com/collections/resveratrol/products/red-wine-resveratrol-tablet
**24.**	Hyalogic Resveratrol Plus	30 Capsules	1 Capsule(s)	20 mg	Hyalogic	https://hyalogic.com/product/resveratrol-plus/
**25.**	Hyalogic Synthovial SEVEN® Plus	30 mL	1 mL	30 mg	Hyalogic	https://hyalogic.com/product/synthovial-seven-plus/

**26.**	Indiana Botanic Gardens Resveratrol 100 mg	30 Softgels	1 Softgel(s)	100 mg	Indiana Botanic Gardens Inc.	https://vite.com/resveratrol-100-mg-indiana-botanic-gardens?v=JqkfZGWqMbz
**27.**	Integrative Therapeutics Resveratrol Ultra	60 Veg Capsules	2 Capsule(s)	125 mg	Integrative Therapeutics LLC.	https://www.integrativepro.com/products/resveratrol-ultra
**28.**	Integrative Therapeutics Resveratrol Ultra HP	60 Softgels	1 Softgel(s)	175 mg	Integrative Therapeutics LLC.	https://www.integrativepro.com/products/resveratrol-ultra-hp
**29.**	Jarrow Formulas Resveratrol	60 Veggie Cap(s)	1 Capsule(s)	100 mg	Jarrow Formulas, Inc	https://jarrow.com/products/resveratrol-60-veggie-caps
**30.**	Jarrow Formulas Resveratrol	120 Veggie Capsules	1 Capsule(s)	100 mg	Jarrow Formulas, Inc	https://jarrow.com/products/resveratrol-120-veggie-caps?_pos=5&_sid=84c85a1c6&_ss=r
**31.**	Jarrow Formulas Pterostilbene	60 Veggie Capsules	1 Capsule(s)	50 mg	Jarrow Formulas, Inc	https://jarrow.com/products/pterostilbene-50-mg-60-veggie-caps?_pos=3&_sid=84c85a1c6&_ss=r
**32.**	Jarrow Formulas Resveratrol Synergy®	60 Tablets	1 Tablet(s)	20 mg	Jarrow Formulas, Inc	https://jarrow.com/products/resveratrol-synergy-60-tablets?_pos=2&_sid=84c85a1c6&_ss=r
**33.**	Life Extension Optimized Resveratrol	60 Vegetarian Capsules	1 Vegetarian Capsule(s)	250 mg	Quality Supplements and Vitamins Inc.	https://www.lifeextension.com/vitamins-supplements/item02230/optimized-resveratrol
**34.**	Life Extension Resveratrol	60 Vegetarian Capsules	1 Vegetarian Capsule(s)	100 mg	Quality Supplements and Vitamins Inc.	https://www.lifeextension.com/vitamins-supplements/item02210/resveratrol
**35.**	Life Extension NAD+ Cell	30 Vegetarian	1 Vegetarian	300	Quality	https://www.lifeextension.com/search#q=res

	Regenerator™ and Resveratrol	Capsules	Capsule(s)	mg	Supplements and Vitamins Inc.	veratrol&t=coveo4A2453FD
**36.**	Life Extension Triple Action Cruciferous Vegetable Extract and Resveratrol	60 Vegetarian Capsules	1 Vegetarian Capsule(s)	20 mg	Quality Supplements and Vitamins Inc.	https://www.lifeextension.com/vitamins-supplements/item01469/triple-action-cruciferous-vegetable-extract
**37.**	Life Extension Grapeseed Extract	60 Vegetarian Capsules	1 Vegetarian Capsule(s)	20 mg	Quality Supplements and Vitamins Inc.	https://www.lifeextension.com/vitamins-supplements/item02211/grapeseed-extract
**38.**	Metabolic Maintenance Resveratrol with Piperine	60 Capsules	1 Capsule(s)	200 mg	Metabolic Maintenance	https://www.metabolicmaintenance.com/Antioxidants/Resveratrol-with-Piperine/
**39.**	Moss Nutrition Resveratrol Select 60 Vc	60 Vegetarian Capsules	1 Capsule(s)	225 mg	Moss nutrition	https://www.mossnutrition.com/product/resveratrol-select-60-vc-m141/
**40.**	Natural Factors ResveratrolRich®	60 Vegetarian Capsules	1 Capsule(s)	250 mg	Natural Factors Nutritional Products Ltd.	https://naturalfactors.com/en-ca/product/resveratrolrich/
**41.**	Natural Factors Resveratrol Rich®	60 Vegetarian Capsules	1 Capsule(s)	500 mg	Natural Factors Nutritional Products Ltd.	https://naturalfactors.com/en-us/product/resveratrolrich-super-strength-500-mg/
**42.**	Nature's Answer resveratrol reserve liquid	5 fl. Oz.	1 Teaspoon(s)	125 mg	Nature's Answer	https://www.naturesanswer.com/product/resveratrol-reserve-liquid/
**43.**	Nature's Answer Resveratrol	60 capsules	1 Capsule(s)	637 mg	Nature's Answer	https://in.iherb.com/pr/nature-s-answer-resveratrol-637-mg-60-vegetarian-capsules/39733
**44.**	Nature's Origin Refined	30 Rapid	1 Capsule(s)	500	Nature's	https://aruba.desertcart.com/products/133385

	Harvest Resveratrol	Release Capsules		mg	Origin	56-natures-origin-refined-harvest-resveratrol-amp-pycnogenol-30-rapid-release-capsules
**45.**	Nature's Plus AgeLoss rejuvabolic - liquid	30 fl. Oz.	2 Tablespoon(s)	250 mg	Natural Organic Laboratories Inc.	https://vitanetonline.com/description/NP80241/vitamins/AGELOSS-REJUVABOLIC/
**46.**	Natures Plus AgeLoss Resveratrol Anti-Aging Complex Bi-Layered Tablets	90 Bi-Layered Tablets-30	3 Bi-Layered Tablet(s)	250 mg	Natural Organic Laboratories Inc.	https://naturesplus.com/products/ageloss-rejuvabolic-resveratrol-anti-aging-complex-bi-layered-tablets
**47.**	Natures Plus Herbal Actives Resveratrol Extended Release Tablets	60 Tablets	2 Extended-Release Tablet(s)	125 mg	Natural Organic Laboratories Inc.	https://naturesplus.com/products/herbal-actives-resveratrol-extended-release-tablets?_pos=1&_sid=27f134572&_ss=r
**48.**	NeoCell Resveratrol Antioxidant	150 Capsules	5 Capsule(s)	100 mg	NeoCell USA	https://www.indiamart.com/proddetail/neocell-resveratrol-antioxidant-150-capsules-13483337062.html
**49.**	NewtonEverett striagen-ds (anti-wrinkle)	30 Capsules	2 Capsules	10 mg	NEWTON-EVERETT®	https://www.newton-everett.com/products/striagen-ds-anti-wrinkle-60-capsules-us?_pos=1&_sid=6bce85d88&_ss=r&variant=40195754918064
**50.**	Newton Everett Biotech Resweratrol & Czerwone Wino Ekstrakt	60 Capsules	1 Capsules	260 mg	NEWTON-EVERETT®	https://www.ceneo.pl/41577384
**51.**	Nora Ross Resveratrol Plus	60 Capsules	2 Capsule(s)	52.5 mg	Nora Ross Inc.	https://noraross.com/products/resveratrol-plus-60-capsules
**52.**	NOW Natural Resveratrol	60 Veg	1 Veg	100	NOW®	https://www.nowfoods.com/supplements/nat

	Veg Capsules Cardiovascular Support*	Capsules	Capsule(s)	mg	Foods	ural-resveratrol-veg-capsules
**53.**	NOW Natural Resveratrol 200 mg Veg Capsules Cardiovascular Support*	120 Veg Capsules	1 Veg Capsule(s)	200 mg	NOW® Foods	https://www.nowfoods.com/supplements/natural-resveratrol-200-mg-veg-capsules
**54.**	NOW Natural Resveratrol 200 mg Veg Capsules Cardiovascular Support*	60 Veg Capsules	1 Veg Capsule(s)	200 mg	NOW® Foods	https://www.nowfoods.com/supplements/natural-resveratrol-200-mg-veg-capsules
**55.**	NOW Resveratrol, Liquid Concentrate	16 fl. Oz.	1 Tablespoon(s)	150 mg	NOW® Foods	https://in.iherb.com/pr/now-foods-resveratrol-liquid-concentrate-16-fl-oz-473-ml/17373
**56.**	NOW Resveratrol, Extra Strength 350 mg Veg Capsules Cardiovascular Support*	60 Veg Capsules	1Veg Capsule(s)	350 mg	NOW® Foods	https://www.nowfoods.com/supplements/resveratrol-extra-strength-350-mg-veg-capsules
**57.**	NutraBio trans-Resveratrol	150 Veg Capsules	1 Vegetable Capsule(s)	250mg	NutraBio Labs Inc.	https://www.nutrabio.com/Products/resveratrol.htm
**58.**	OL Olympian Labs Trans-Resveratrol 150 Mg Vegetarian and Kosher Capsules	30 Vegetarian Capsules	1 Capsule(s)	75 mg	OL Olympian Labs	https://www.heb.com/product-detail/olympian-labs-clinical-trans-resveratrol-150-mg-vegetarian-and-kosher-capsules/1479253
**59.**	OL Olympian Labs Clinical Trans-Resveratrol,	60 Vegetarian Capsules	1 Capsule(s)	500 mg	OL Olympian Labs	https://www.amazon.in/Olympian-Labs-Clinical-Trans-Resveratrol-Packaging/dp/B002WTC5HS
**60.**	Physician's Preference Resveratrol with Red Wine Extract - 200 m	60 Vegetarian Capsules	1 Vcap(s)(R)	200 mg	Physician's Preference	https://kusoglife.com/products/physicians-preference-resveratrol-with-red-wine-extract-200-mg/
**61.**	Planetary Herbals Resveratrol Extract with	30 Tablets	2 Tablet(s)	80 mg	Planetary Herbals LLC.	https://www.planetaryherbals.com/products/GP1895

	Red Wine Tablets					
**62.**	Planetary Herbals Resveratrol Extract with Red Wine Tablets	60 Tablets	2 Tablet(s)	80 mg	Planetary Herbals LLC.	https://www.amazon.in/Planetary-Herbals-Resveratrol-Extract-Tablets/dp/B000QB7NXA
**63.**	Pomology 300 mg Anti-Aging Supplement With Resveratrol	60 Vegetarian Capsules	2 Capsule(s)	300 mg	-	https://www.instacart.com/products/18737988-pomology-300-mg-anti-aging-supplement-with-resveratrol-60-ct
**64.**	Prescribed Choice Super Strength Researched Trans Resveratrol Capsules, 30 Count	30 Vegetarian Capsules	1 Capsule(s)	250 mg	Prescribed Choice	https://www.amazon.in/Prescribed-Choice-Strength-Researched-Resveratrol/dp/B0083UXLL4
**65.**	ProCaps Laboratories Resveratrol-100™ with Green Tea 100 Grape Seed 100 Pomegranate 100 - Capsules | Anti-oxidants	360 Easy-To-Swallow Capsules Size-30	1 Capsule(s)	100 mg	ProCaps Laboratories	https://www.procapslabs.com/Products/Details/268447362/Resveratrol-100-with-Green-Tea-100-Grape-Seed-100-Pomegranate-100
**66.**	ProCaps Laboratories Women's Elite-100™ - Packets | Multivitamins	360 Easy-To-Swallow Capsules Size-60	1 Capsule(s)	100 mg	ProCaps Laboratories	https://www.procapslabs.com/Products/Details/268447696/Multivitamin-Women-s-Elite-100
**67.**	ProCaps Laboratories Resveratrol-100™ - Capsules | Anti-oxidants	360 Easy-To-Swallow Capsules	1 Capsule(s)	100 mg	ProCaps Laboratories	https://www.procapslabs.com/Products/Details/268435830/Resveratrol-100#Label
**68.**	ProCaps Laboratories Fruit Full Anti-Oxidant Extracts™ - Capsules | Anti-oxidants	Easy-To-Swallow Capsules	1 Capsule(s)	20 mg	ProCaps Laboratories	https://www.procapslabs.com/Products/Details/268447158/Fruit-Full-Anti-Oxidant-Extracts
**69.**	Protocol For Life Balance Resveratrol	60 Veg Capsules	1 Veg Capsule(s)	200 mg	Protocol For Life Balance	protocolforlife.com/resveratrol-200-mg/

**70.**	Protocol For Life Balance Glycogen synthase kinase-3, resveratrol, curcumin, berberine	90 Veg Capsules	1 Veg Capsule(s)	-	Protocol For Life Balance	https://www.protocolforlife.com/glucose-management-with-berberine-hcl/
**71.**	Pure Encapsulations Resveratrol VESIsorb® 90's	90 Caplique Capsules	2 Caplique Capsule	100 mg	Pure Encapsulations, LLC.	https://www.pureencapsulationspro.com/resveratrol-vesisorb.html
**72.**	PureBulk.com trans-resveratrol powder pure bulk 5kg	10 Grams	250 mg	-	PureBulk	https://purebulk.com/products/resveratrol-pure-bulk
**73.**	PurHEALTH Trans-Resveratrol by ProHealth Longevity	60 Capsules	3 Capsule(s)	1000 mg per 2 capsule serving	PurHEALTH Inc.	https://www.prohealthlongevity.com/products/prohealth-trans-resveratrol-1000-mg-60-capsules-ph528
**74.**	Puritan's Pride Premium Resveratrol 250 mg plus Red Wine Extract	60 Rapid Release Softgels	1 Softgel(s)	250 mg	Puritan's Pride Inc.	https://www.puritan.com/resveratrol-053/resveratrol-250-mg-027980
**75.**	Reserveage Nutrition Resveratrol 500mg Sustained Release	60 Veggie Capsules	1 Veggie Capsule(s)	500mg	Twinlab Consolidated	https://reserveage.tlcchealth.com/products/resveratrol-500mg
**76.**	Reserveage Organics Liquid Resveratrol Super Berry	5 fl. Oz.	1 Teaspoon(s)	125 mg		https://www.evitamins.com/in/liquid-resveratrol-reserveage-organics-85041
**77.**	ResVitale Resveratrol 500	60 Vegetarian	2 Vegetarian	800	GNC	https://www.gnc.com/resveratrol/446813.ht

	mg	Capsules	Capsules	mg	Holdings, LLC	ml#q=resveratrol&lang=default&start=1
**78.**	ResVitale Revitalizing Fruit Chews	30 Softchews	1 Chew(s)	80 mg	GNC Holdings, LLC	https://www.gnc.com/resveratrol/446748.html#q=resveratrol&lang=default&start=1
**79.**	Seeking Health Optimal Liposomal Curcumin with Resveratrol	60 Vegetarian Capsules	1 Capsule(s)	75 mg	Seeking Health.	https://www.seekinghealth.com/products/optimal-liposomal-curcumin-with-resveratrol-30-servings
**80.**	Solaray Resveratrol Supplement	60 Vegetarian Capsules	1 Vegetarian Capsule(s)	75 mg	Solaray	https://www.amazon.in/Solaray-Resveratrol-Supplement-75-Count/dp/B00028OQL4
**81.**	Solgar resveratrol 100 mg vegetable capsules	60 Vegetable Capsules	1 Vegetable Capsule(s)	100 mg	Solgar Inc.	https://www.solgar.com/products/resveratrol-100-mg-vegetable-capsules/
**82.**	Solgar resveratrol 250 mg with red wine extract softgels	30 Softgels	1 Softgel(s)	250 mg	Solgar Inc.	https://www.solgar.com/products/resveratrol-250-mg-with-red-wine-extract-softgels/
**83.**	Spring Valley Spring Valley Resveratrol Plus Red Wine Extract Softgels,	30 Softgels	1 Softgel(s)	250mg	Spring Valley	https://www.amazon.in/Spring-Valley-Resveratrol-Extract-Softgels/dp/B00DJM45Y8
**84.**	Sunshine Naturals Resveratrol with Co Q10 & Hawthorne Berries	90 Capsules	2 Capsule(s)	200 mg	Sunshine Naturals	https://kusoglife.com/products/sunshine-naturals-resveratrol-with-co-q10-and-hawthorne-berries/
**85.**	Swanson_Ultra- Resveratrol	30 Capsules	1 Capsule(s)	500 mg	Swanson	https://www.swansonvitamins.com/swanson-ultra-resveratrol-500-500-mg-30-caps
**86.**	Swanson Ultra Resveratrol & Quercetin with Grape Seed Extract - 3 in 1 Formula	30 Veggie Capsules	1 Veggie Capsule(s)	100 mg	Swanson	https://www.swansonvitamins.com/swanson-ultra-resveratrol-quercetin-30-veg-caps
**87.**	Swanson Ultra	30 Capsules	1 Capsule(s)	250	Swanson	https://www.swansonvitamins.com/swanson-

	Resveratrol - Higher Potency			mg		ultra-resveratrol-250-250-mg-30-caps
**88.**	Swanson Ultra Resveratrol Complex	60 Capsules	1 Capsule(s)	180 mg	Swanson	https://www.swansonvitamins.com/swanson-ultra-resveratrol-complex-60-caps
**89.**	The Vitamin Shoppe Reservie Trans-Resveratrol Antioxidant - Healthy Aging -	60 Veggie Capsules	1 Veggie Capsule(s)	250 mg	The Vitamin Shoppe Inc.	https://www.vitaminshoppe.com/p/Reservie-Trans-Resveratrol-250-MG-60-Veggie-Caps/vs-3554
**90.**	Vinco's Resveratrol with Red Wine Extract	60 Softgels	1 Softgel(s)	250 mg	Vinco's	https://kusoglife.com/products/vincos-resveratrol-with-red-wine-extract/
**91.**	Vitabase Enfūz (Advanced)	60 Capsules	2 Capsule(s)	20 mg	Vitabase	https://vitabase.com/product/enfuz-advanced/
**92.**	Vital Nutrients Resveratrol 500mg Supplement	60 Vegetarian Capsules	1 Vegetarian Capsule(s)	500 mg	Vital Nutrients	vitalnutrients.net/resveratrol.html
**93.**	Vital Nutrients Hormone Balance	60 Vegetarian Capsules	1 Vegetarian Capsule(s)	10 mg	Vital Nutrients	https://www.vitalnutrients.net/hormone-balance.html
**94.**	Vitamer Laboratories Resveratrol	60 Vegetarian Capsules	1Capsule(s)	150 mg	Vitamer Laboratories.	http://www.vitamer.com/search?search=resveratrol
**95.**	Vitamin Research Products Extension Resveratrol 300 mg	90 Capsules	1 Capsule(s)	300 mg	VRP LLC.	https://kusoglife.com/products/vitamin-research-products-extension-resveratrol-300-mg/
**96.**	Vitamin WorldMaximum Strength Resveratrol	60 Easy to Swallow Coated Caplets	1 Softgel(s)	500 mg.	Vitamin World USA Corporation	https://www.vitaminworld.com/resveratrol-500-mg-0070031043.html
**97.**	VSN Vital Strength Nutrition Resveratrol 150 mg	60 Vegetarian Capsules	1 Capsule(s)	150 mg	Vital Strength Nutrition	https://kusoglife.com/products/vsn-vital-strength-nutrition-resveratrol-150-mg/
**98.**	Wonder Laboratories	120 Softgels	1 Softgel(s)	50 mg	Wonder	https://www.wonderlabs.com/itemleft.php?it

	Resveratrol 50 mg | Japanese Knotweed				Laboratories	emnum=5061
**99.**	Youtheory resveratrol	160 Tablets	4 Tablet(s)	250 mg	Nutrawise Irvine, USA	https://kusoglife.com/products/youtheory-resveratrol-1/
**100.**	Zhou Resveratrol	30 capsules	2 vegetable capsules	1000 mg	Zhou Nutrition, park city	https://www.Zhounutrition.com/products/resveratrol
